# Observation of orbital-angular-momentum-driven temperature modulation via the spin Peltier effect

**DOI:** 10.1126/sciadv.aej1838

**Published:** 2026-10-02

**Authors:** Sang J. Park, Tsuneyoshi Kan, Takamasa Hirai, Ken-ichi Uchida

**Affiliations:** ^1^ National Institute for Materials Science, Tsukuba 305-0047, Japan.; ^2^ Department of Advanced Materials Science, Graduate School of Frontier Sciences, The University of Tokyo, Kashiwa 277-8561, Japan.

## Abstract

Angular-momentum transport provides a pathway for controlling energy flow in solids beyond conventional charge-based mechanisms. Although spin currents are known to mediate spin-caloritronic phenomena such as the spin Peltier effect (SPE), the role of orbital angular momentum (OAM) in heat transport remains largely unexplored. Here, we demonstrate OAM-driven temperature modulation via the SPE in yttrium-iron-garnet/Pt/CuO*_x_* heterostructures. Using wedge-shaped CuO*_x_* layers combined with spatially resolved active thermal measurement techniques, we map the continuous thickness dependence and quantitatively disentangle the spin- and orbital-current-mediated contributions within a single device. The orbital-mediated component exhibits a pronounced maximum at an intermediate thickness, revealing a characteristic length scale for interfacial OAM generation and propagation at the Cu/CuO*_x_* interface. These results provide direct experimental evidence that charge-current-driven OAM can drive SPE-induced temperature modulation, establishing interfacial orbital processes as an additional channel for heat transport and providing a pathway toward spin-orbit caloritronics.

## INTRODUCTION

Controlling the flow of energy in solids is central to next-generation information and energy technologies (*1*, *2*). Beyond conventional charge and phonon transport, angular momentum has emerged as a fundamental degree of freedom governing nonequilibrium dynamics in condensed matter systems. In spintronics, the generation and manipulation of spin currents, or fluxes of spin angular momentum, enable electrical control of magnetism through spin-orbit coupling (SOC) (*3*, *4*), thereby establishing angular momentum as an active channel for controlling material functionality.

When coupled with heat, angular-momentum dynamics give rise to spin-caloritronic phenomena (*5*–*7*). Representative examples include the spin Seebeck effect (SSE) (*8*, *9*) and the spin Peltier effect (SPE) (*10*, *11*), which are related through Onsager reciprocity and enable interconversion between heat and spin currents at magnetic interfaces. These discoveries have established spin angular momentum as a key mediator of temperature modulation in magnetic heterostructures. To date, most experimental realizations rely on heavy metals (HMs) with strong SOC such as Pt (*12*, *13*), reflecting a spin-centric framework in which spin currents are assumed to be the primary carriers of thermo-spin conversion.

Recent advances in orbitronics challenge this spin-centric paradigm by identifying orbital angular momentum (OAM) as a distinct transport channel in solids (*14*–*23*). The orbital Hall effect (OHE) (*16*–*18*) and orbital Rashba-Edelstein effect (OREE) (*19*–*21*) demonstrate that charge currents can generate orbital transport in bulk materials and orbital accumulation at inversion-symmetry-broken interfaces, respectively. Theoretically, such orbital responses originate from momentum-space orbital textures and are predicted to be widespread in solids, often exceeding their spin counterparts in magnitude (*14*, *24*). In materials with finite SOC, these orbital responses can be partially converted into spin currents through bulk or interfacial spin-orbit interactions (*16*, *18*), providing a pathway for orbital-to-spin angular-momentum conversion.

Although orbital transport has been studied mainly in the context of magnetic switching and torque generation (*15*, *25*, *26*), recent studies have begun to explore its role in thermally driven transport. Theoretical studies have predicted thermally driven orbital transport phenomena in magnetic systems (*27*, *28*). Experimentally, the orbital Seebeck effect, an orbital counterpart of the SSE, has been demonstrated in ferrimagnetic insulators through thermal magnons (*29*) and in nonmagnetic insulators through chiral phonons (*30*). However, whether charge-current-driven OAM can induce temperature modulation via the SPE remains unexplored.

Here, we report the observation of OAM-driven temperature modulation via the SPE in ferromagnetic or ferrimagnetic insulator (FMI)/HM/orbital-active material (OM) heterostructures (Fig. 1). Using yttrium-iron-garnet (YIG; FMI)/Pt (HM)/naturally oxidized Cu (CuO*_x_*; OM) trilayers, we observe a pronounced temperature modulation associated with the introduction of the CuO*_x_* layer. By continuously tuning the CuO*_x_* thickness (*t*_CuO*x*_ = 0 to 16 nm) in wedge-shaped structures and spatially resolving the resulting temperature modulation using lock-in thermography (LIT), we identify a well-defined thickness at which the temperature modulation is maximized. At this optimal thickness, the orbital-current-mediated contribution reaches up to ∼3 times the conventional spin-Hall-driven SPE response in Pt, demonstrating efficient charge-to-heat conversion mediated by OAM. The decrease in modulation efficiency with increasing *t*_CuO*x*_ indicates that interfacial coupling involving the CuO*_x_* layer plays a key role in the observed behavior, supporting an interfacial origin associated with the OREE. These findings provide direct evidence that interfacial OAM generation and transport contribute to SPE-induced temperature modulation, thereby extending the framework of spin-orbit caloritronics beyond conventional spin-dominated paradigms.

**Fig. 1.**
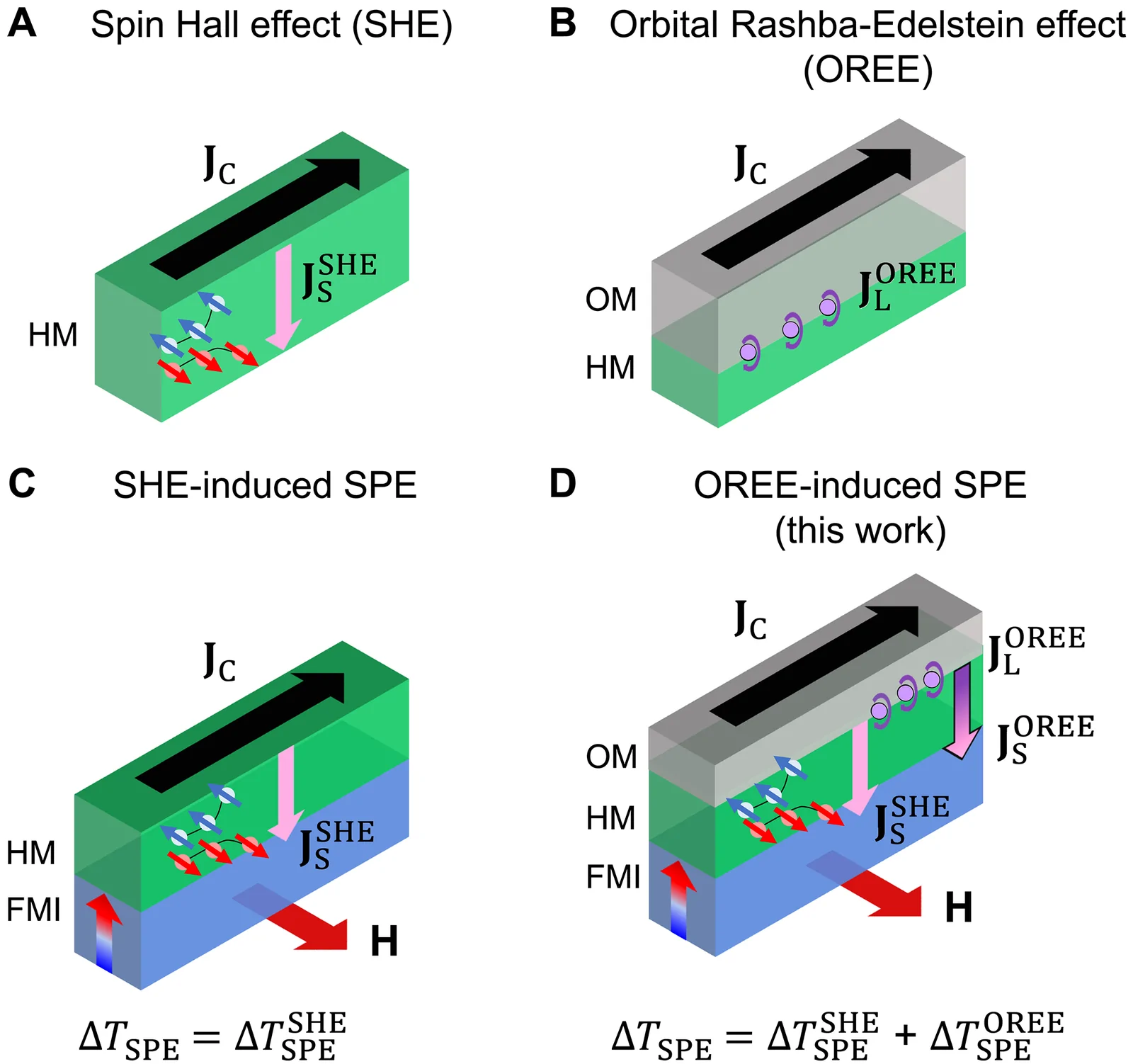
Conceptual illustration of spin- and orbital-angular-momentum-driven temperature modulation via the SPE. (**A**) Generation of a spin current $J_{S}^{\text{SHE}}$ via the SHE in an HM with large SOC. (**B**) Generation of interfacial OAM, followed by orbital-current propagation $J_{L}^{\text{OREE}}$, at the HM/OM interface with broken inversion symmetry via the OREE. (**C**) Temperature modulation through the conventional SPE ($∆T_{\text{SPE}}$) in a FMI/HM bilayer, driven solely by the spin current $J_{S}^{\text{SHE}}$ (i.e., $∆T_{\text{SPE}}=∆T_{\text{SPE}}^{\text{SHE}}\propto J_{S}^{\text{SHE}}$). (**D**) Orbital-angular-momentum-driven SPE in an FMI/HM/OM trilayer, driven by both $J_{S}^{\text{SHE}}$ and the orbital current $J_{L}^{\text{OREE}}$. The $J_{L}^{\text{OREE}}$ is converted into a spin current $J_{S}^{\text{OREE}}$ in the HM via SOC. The resulting temperature modulation is given by $∆T_{\text{SPE}}=∆T_{\text{SPE}}^{\text{SHE}}+∆T_{\text{SPE}}^{\text{OREE}}$, with $∆T_{\text{SPE}}^{\text{OREE}}\propto J_{S}^{\text{OREE}}\propto J_{L}^{\text{OREE}}$.

## RESULTS

### Experimental system

Figure 1 illustrates the conceptual framework of the present study. In a conventional FMI/HM bilayer, a charge current **J**_C_ flowing in the HM generates a transverse spin current $J_{S}^{\text{SHE}}$ via the spin Hall effect (SHE) (Fig. 1A) (*3*). Subsequently, the injected spin current exchanges angular momentum with magnons at the FMI/HM interface, producing interfacial heat absorption or release through the SPE (Fig. 1C) (*10*, *11*).

To examine whether orbital currents can similarly drive angular-momentum transfer responsible for temperature modulation, we introduced an OM layer on top of the HM, forming FMI/HM/OM trilayers (Fig. 1D). At interfaces involving this layer, inversion symmetry breaking enables the generation of nonequilibrium OAM via the OREE (Fig. 1B), an orbital analog of the Rashba-Edelstein effect (*31*, *32*). The generated OAM can propagate toward the HM as an orbital current $J_{L}^{\text{OREE}}$, where it is subsequently converted into a spin current $J_{S}^{\text{OREE}}$ through SOC, thereby providing an additional contribution to interfacial angular-momentum injection and the resulting SPE alongside the conventional $J_{S}^{\text{SHE}}$.

To experimentally implement this concept, we used YIG, Pt, and CuO*_x_* as the FMI, HM, and OM layers, respectively. To systematically examine the orbital contribution, we fabricated a wedge-shaped CuO*_x_* layer with continuously varying thickness (*t*_CuO*x*_) on a uniform YIG/Pt bilayer (Fig. 2 and Materials and Methods), covering a *t*_CuO*x*_ range of 0 to 16 nm. This geometry enables *t*_CuO*x*_-dependent evaluation without batch-to-batch variations in thin-film growth and measurement conditions. Because the CuO*_x_* layer is formed through natural oxidation of deposited Cu, the layer consists of coexisting metallic and oxidized Cu regions with a *t*_CuO*x*_-dependent distribution, which is expected to promote OAM generation at the Cu/CuO*_x_* interface (*15*, *20*, *33*–*35*). All uncapped samples were exposed to ambient air for 2 days, after which electrical contacts were formed using silver epoxy, which was cured at 80°C for 30 min before the LIT measurements. A time-dependent resistance measurement using the same processing sequence showed a rapid initial resistance increase during ambient exposure and a further change following the 80°C curing treatment (fig. S1). Throughout this manuscript, we refer to this mixed Cu/CuO*_x_* structure as an effective OM layer and denote it as CuO*_x_*. Accordingly, *t*_CuO*x*_ represents the total thickness of the layers containing both metallic and oxidized Cu regions.

**Fig. 2.**
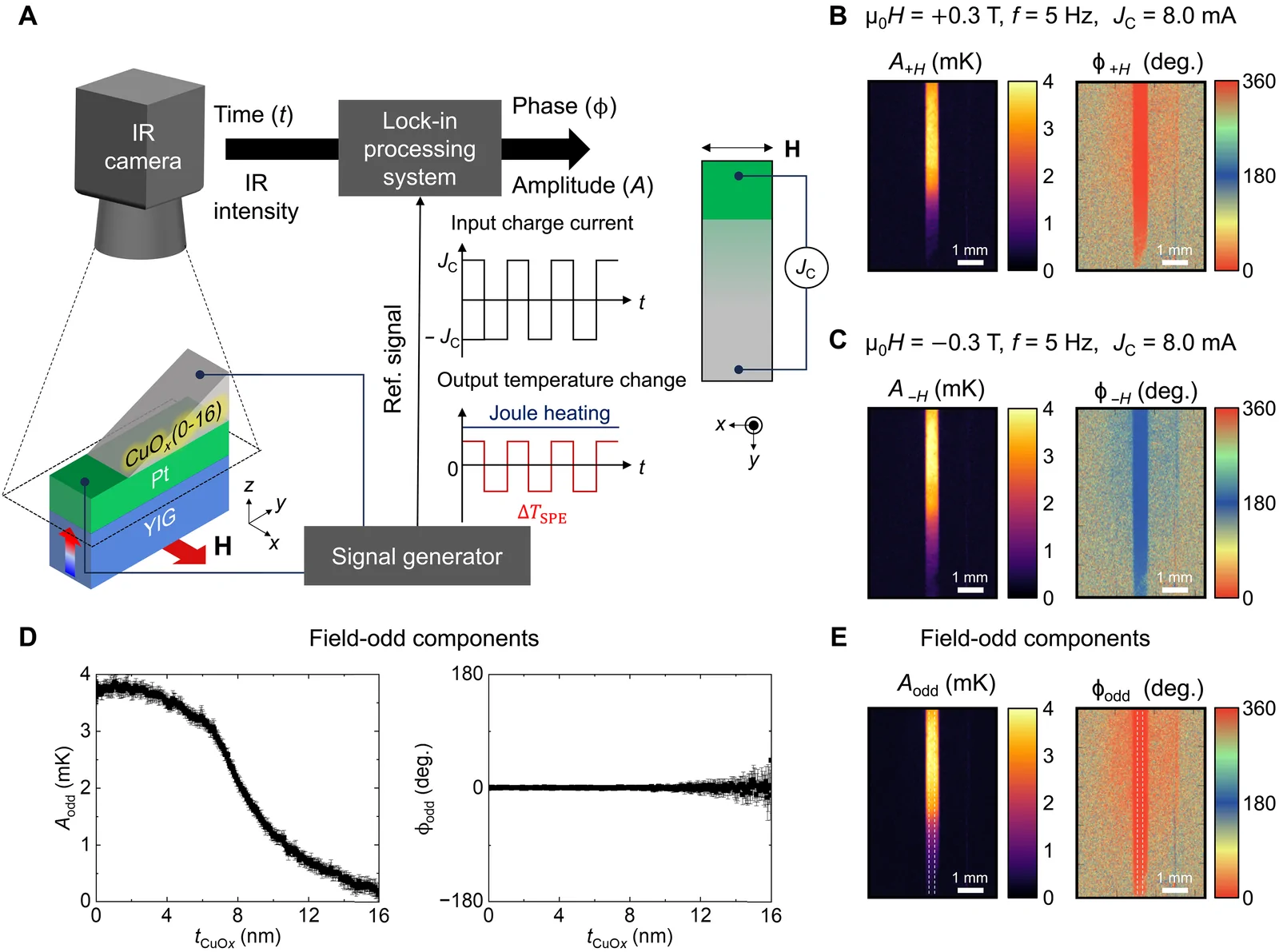
Observation of angular-momentum-driven temperature modulation via the SPE using LIT. (**A**) Observation of SPE-induced temperature modulation using LIT in a CuO*_x_* wedge layer fabricated on a YIG/Pt bilayer. The CuO*_x_* thickness (*t*_CuO*x*_) was continuously varied from 0 to 16 nm in a single sample, and the signal was spatially resolved using an infrared camera with high spatial resolution and temperature sensitivity. A square-wave-modulated current with zero offset was applied to isolate the SPE-induced temperature modulation (see Materials and Methods). An external magnetic field **H** was applied along the *x* axis to satisfy the symmetry condition for detecting the SPE-induced temperature modulation along the *z* direction using LIT. (**B** and **C**) Measured lock-in amplitude and phase under magnetic fields of (B) μ_0_*H* = +0.3 T and (C) μ_0_*H* = −0.3 T, respectively, at a frequency of *f* = 5 Hz and a current amplitude of *J*_C_ = 8.0 mA. The corresponding quantities are denoted by $A_{+H}$ and $\phi_{+H}$ in (B) and by $A_{-H}$ and $\phi_{-H}$ in (C). The LIT images were acquired using a 1× infrared lens, corresponding to a pixel size of 15 × 15 μm^2^. deg., degrees. (**D**) Line profiles of the field-odd lock-in amplitude *A*_odd_ and phase ϕ_odd_ as functions of *t*_CuO*x*_. The *A*_odd_ and ϕ_odd_ values represent spatial averages along the *x* direction over the region between the dashed lines in (E). The error bars indicate the SD. (**E**) Spatial maps of the field-odd lock-in amplitude *A*_odd_ and phase ϕ_odd_, obtained from complex antisymmetrization of the LIT signals measured in (B) and (C), as defined in Materials and Methods.

We used a 72-μm-thick YIG grown by liquid-phase epitaxy on a gadolinium-gallium-garnet (GGG) substrate (Materials and Methods). This thickness is much larger than both the magnon energy relaxation length (∼250 nm) (*36*, *37*) and the magnon diffusion length (∼10 μm) (*38*) in YIG at room temperature, enabling bulk-like magnetic behavior without boundary or diffusion limitations. Accordingly, the GGG substrate is not explicitly discussed throughout the manuscript. The Pt thickness was fixed at 2.5 nm, which provides robust SOC-mediated angular-momentum conversion within the layer (*18*).

### Thickness-dependent SPE-induced temperature modulation

The SPE-induced temperature modulation was detected using LIT under a square-wave-modulated ac charge current with zero dc offset and a steady magnetic field *H* (Fig. 2A) (*39*, *40*). The infrared camera measured the amplitude *A* and phase ϕ of the temperature oscillation relative to the applied current *J*_C_. To ensure sufficiently high and spatially uniform infrared emissivity, the surfaces of all samples were coated with black ink having an emissivity greater than 0.94 (fig. S2). The patterned line was oriented along the *t*_CuO*x*_-gradient direction (*y*), enabling continuous *t*_CuO*x*_-dependent evaluation of the SPE-induced temperature modulation under a constant current density *j*_C_. The measurements were performed at a modulation frequency of *f* = 5 Hz and opposite saturating magnetic fields of μ_0_*H* = ±0.3 T, where μ_0_ is the vacuum permeability.

Figure 2 (B and C) shows the *A* and ϕ images measured at μ_0_*H* = +0.3 and −0.3 T, respectively. Upon reversal of the magnetic field, the lock-in amplitude remained similar, whereas the phase in the current-carrying region changed by ∼180°, indicating reversal of the complex temperature-modulation signal with the YIG magnetization. To extract the magnetization-dependent, field-odd component associated with spin and/or OAM transfer, the complex LIT signals were antisymmetrized according to $Z_{\text{odd}}=\left[Z\left(+H\right)-Z\left(-H\right)\right]/2$ (see Materials and Methods). Because Joule heating (∝ *J*_C_^2^) yields a constant background without a first-harmonic response and the conventional Peltier effect (∝ *J*_C_) is field independent, this procedure isolates the SPE-induced temperature modulation that is linear in *J*_C_ and odd with respect to magnetization (*11*, *41*).

Figure 2E shows the resulting spatial maps of the field-odd lock-in amplitude *A*_odd_ and phase ϕ_odd_, obtained from the raw signals shown in Fig. 2 (B and C). The LIT images had a pixel size of 15 × 15 μm^2^, corresponding to an effective *t*_CuO*x*_ sampling interval of 0.04154 nm per pixel along the wedge gradient. The corresponding *t*_CuO*x*_-dependent profiles shown in Fig. 2D were obtained by averaging the spatial maps along the constant-*t*_CuO*x*_ direction (*x* direction). The field-even components were negligibly small, as shown in fig. S3.

Figure 2D shows that *A*_odd_ remains nearly constant up to *t*_CuO*x*_ ∼ 4 nm, followed by a gradual and monotonic decrease with increasing *t*_CuO*x*_. The corresponding ϕ_odd_ remains stable within the finite-signal regime and becomes noisy only when *A*_odd_ approaches the sub-millikelvin regime at larger *t*_CuO*x*_. No systematic phase shift or sign reversal is observed over the entire finite-signal regime. The constant ϕ_odd_ suggests that the SPE contributions associated with Pt and CuO*_x_* share the same sign and symmetry with respect to magnetization and charge-current direction, consistent with previous observations in TmIG/Pt/CuO*_x_* systems (*15*). The systematic suppression of *A*_odd_ at larger *t*_CuO*x*_ suggests a reduction of the effective angular-momentum injection at the YIG/Pt interface, potentially associated with (i) current redistribution within the multilayer and/or (ii) modification of interfaces involving the CuO*_x_* layer responsible for OAM generation.

To further characterize the magnetic-field and current dependences of the temperature modulation, we performed additional LIT measurements using an isolated YIG/Pt(2.5 nm)/CuO*_x_*(5.6 nm) device. *A* and ϕ were measured at magnetic-field magnitudes ranging from 0.08 to 0.30 T under both field polarities. Within the saturated-field regime, the amplitude remained nearly constant within the experimental uncertainty, whereas the phase reversed with the magnetic-field polarity (fig. S4). These results show that the temperature-modulation signal follows the YIG magnetization orientation. In addition, current-dependent measurements showed that *A*_odd_ increased linearly with *J*_C_ (*R*^2^ > 0.9999) (fig. S5). The field-odd symmetry and linear current dependence support the assignment of the observed field-odd temperature modulation to the SPE and distinguish it from the field-independent conventional Peltier effect and *J*_C_^2^-dependent Joule heating contribution. Because the observed temperature modulation contains contributions from both the conventional SHE in Pt and the orbital-mediated component associated with CuO*_x_*, quantitative separation of these contributions is required to clarify the role of OAM in SPE-induced temperature modulation.

### Quantitative separation of spin- and orbital-current contributions

To clarify the orbital contribution, we quantitatively separated the individual components associated with the SHE and orbital-current mechanisms based on the thickness-dependent current distribution within the multilayer. We first evaluated the effective electrical resistivity ρ of the CuO*_x_* films as a function of *t*_CuO*x*_ using a standard four-probe method (Materials and Methods and Fig. 3A, inset). As mentioned above, *t*_CuO*x*_ represents the effective thickness of the Cu/CuO*_x_* layers. The electrical resistivity exhibits strong thickness dependence in the ultrathin regime; ρ increases when *t*_CuO*x*_ decreases because of enhanced interface scattering and reduced metallic conduction thickness. At *t*_CuO*x*_ = 2 nm, ρ increases sharply by ∼1 order of magnitude to ∼1.1 × 10^3^ μΩ·cm, indicating the percolation limit and suggesting an effective oxidation layer thickness of ∼2 to 3 nm.

**Fig. 3.**
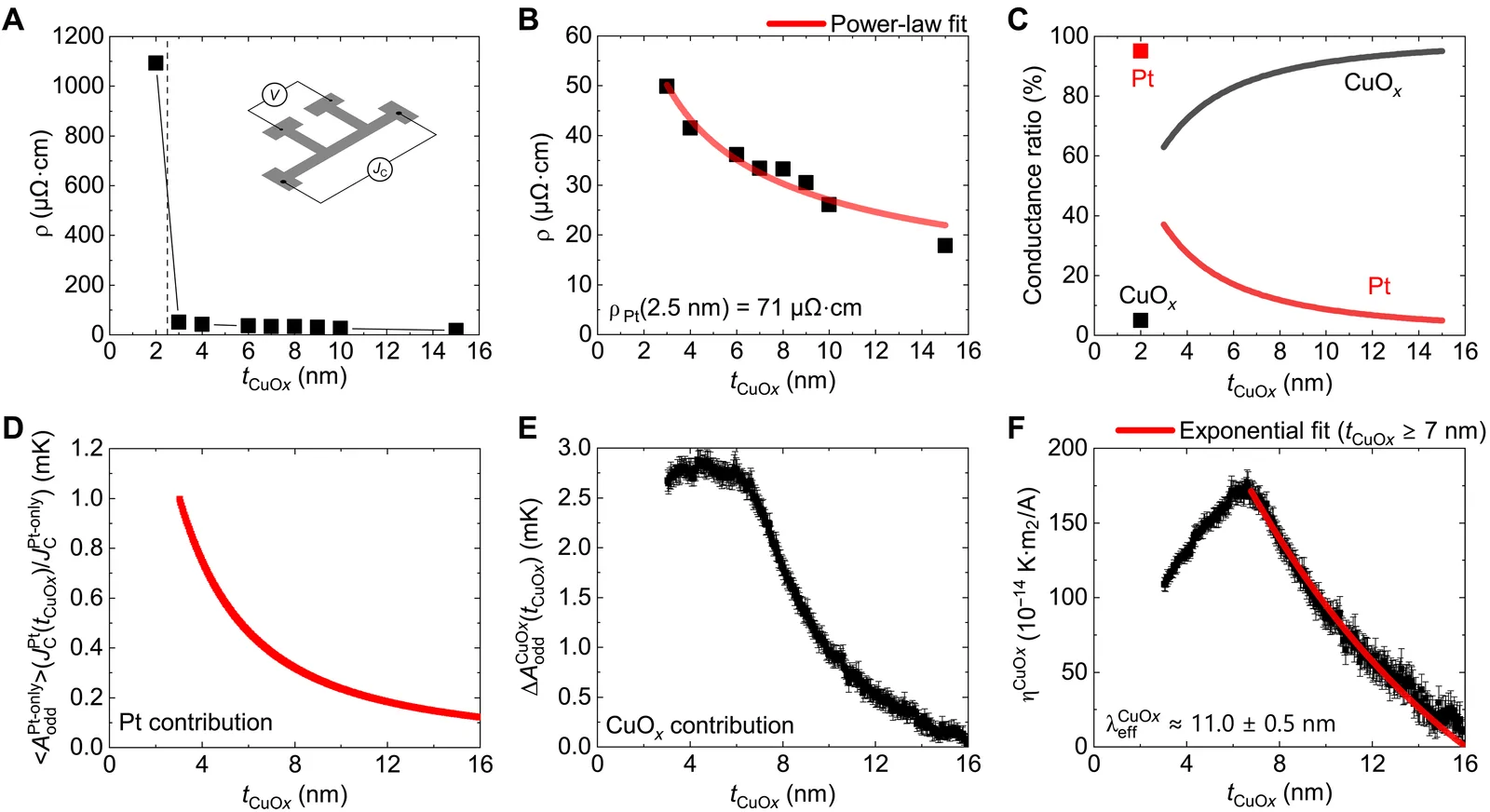
Quantitative separation of SPE contributions from Pt and CuO*_x_* based on bilayer electrical conductance. (**A**) Electrical resistivity ρ of uniform CuO*_x_* films as a function of CuO*_x_* thickness (*t*_CuO*x*_), measured using the standard four-probe method (inset). The dashed line indicates the percolation limit. (**B**) Selected view of ρ for 3- to 15-nm-thick films. The data are fitted using a phenomenological power-law function, $\rho \left(t_{\text{CuO}x}\right)=\rho_{\infty }+C{t_{\text{CuO}x}}^{-n}$, where $\rho_{\infty }$ is the bulk resistivity limit of Cu, and *C* and *n* are fitting parameters. The ρ of 2.5-nm-thick Pt was measured to be 71 μΩ·cm. The error bars for (A) and (B) are smaller than the symbols. (**C**) Electrical conductance ratio of CuO*_x_*(*t*_CuO*x*_) and Pt(2.5 nm) as a function of *t*_CuO*x*_, used to determine the current distribution within the multilayer. (**D** and **E**) Separated components of the measured SPE-induced temperature modulation signal *A*_odd_ as a function of *t*_CuO_*_x_*: (D) the Pt contribution and (E) the orbital contribution associated with CuO*_x_* (see Eq. 2 for details). (**F**) Charge-to-heat conversion efficiency η^CuO*x*^ associated with the CuO*_x_* orbital contribution, defined as SPE-induced temperature modulation per the current density flowing through the CuO*_x_* layer (see Eq. 3). The decay in the *t*_CuO*x*_ ≥ 7 nm region is fitted using an exponential function $\text{exp}\left(-\frac{t_{\text{CuO}x}}{\lambda_{\text{eff}}^{\text{CuO}x}}\right)$. The values in (F) are replotted in Fig. 4 and fig. S5C for comparison.

To estimate the current distribution in the multilayers continuously as a function of *t*_CuO*x*_, we described the resistivity for *t*_CuO*x*_ in the range of 3 to 15 nm using a phenomenological power-law form

$$\rho \left(t_{\text{CuO}x}\right)=\rho_{\infty }+C{t_{\text{CuO}x}}^{-n}$$ (1)

where $\rho_{\infty }$ is the bulk resistivity limit of Cu (1.68 μΩ·cm), and *C* and *n* are fitting parameters (Fig. 3B). This fitting allows estimation of the resistivity values for continuous current-distribution analysis in the wedged sample. The fitting parameters are *C* = 88.2 ± 7.9 and *n* = 0.513 ± 0.051. The resistivity of 2.5-nm-thick Pt (ρ_Pt_) was measured independently as 71 μΩ·cm.

Using the extracted resistivity values and layer thickness, we determined the sheet conductance of Pt (*G*_Pt_) and CuO*_x_* (*G*_CuO*x*_) layers and calculated the thickness-dependent conductance ratio. Assuming parallel current flow within the metallic layers, the fractions of the applied current flowing through Pt and CuO*_x_* were determined from their relative conductances as *G*_Pt_/*G*_tot_ and *G*_CuO*x*_/*G*_tot_, where *G*_tot_ = *G*_Pt_ + *G*_CuO*x*_. Figure 3C shows the conductance ratio, which represents the current distribution within the multilayer. At *t*_CuO*x*_ = 3 nm, ∼63% of the applied current flows through the CuO*_x_* layer, and this fraction increases with increasing *t*_CuO*x*_. It is noted that the current flowing through a 2-nm-thick CuO*_x_* layer is merely 5% of the total applied current, suggesting that the SPE signal observed below 2 nm is primarily governed by conventional charge-to-spin conversion processes rather than by orbital transport through the ultrathin CuO*_x_* layer.

On the basis of this conductance model, we separated the SPE-induced temperature modulation attributed to the CuO*_x_* layer by subtracting the Pt-only contribution expected from the conventional SHE. As experimentally observed in Fig. 2, ϕ_odd_ remains constant; therefore, the complex lock-in signals add constructively without additional phase lag, allowing direct subtraction of the amplitudes. Accordingly, the CuO*_x_*-associated component is obtained as

$$∆A_{\text{odd}}^{\text{CuO}x}\left(t_{\text{CuO}x}\right)=\langle A_{\text{odd}}^{\text{Pt}/\text{CuO}x}\left(t_{\text{CuO}x}\right)\rangle -\langle A_{\text{odd}}^{\text{Pt}-\text{only}}\rangle \left[\frac{J_{C}^{\text{Pt}}\left(t_{\text{CuO}x}\right)}{J_{C}^{\text{Pt}-\text{only}}}\right]$$ (2)

where $\langle A_{\text{odd}}^{\text{Pt}/\text{CuO}x}\left(t_{\text{CuO}x}\right)\rangle$ and $\langle A_{\text{odd}}^{\text{Pt}-\text{only}}\rangle$ represent the spatial averages in the constant *t*_CuO*x*_ region (along the *x* direction) and the YIG/Pt reference region, respectively. The Pt contribution (second term on the right-hand side of Eq. 2) is shown in Fig. 3D, whereas the CuO*_x_*-associated component (left-hand side) is presented in Fig. 3E. $J_{C}^{\text{Pt}}\left(t_{\text{CuO}x}\right)$ is the thickness-dependent current flowing through the Pt layer, estimated from the conductance model as $J_{C}^{\text{Pt}}\left(t_{\text{CuO}x}\right)=\left(G_{\text{Pt}}/G_{\text{tot}}\right)J_{C}$ (Fig. 3C). The resulting CuO*_x_*-associated SPE contribution was then normalized by the corresponding current density $j_{C}^{\text{CuO}x}\left(t_{\text{CuO}x}\right)$ flowing through the CuO*_x_* layer as

$$\eta^{\text{CuO}x}=∆A_{\text{odd}}^{\text{CuO}x}\left(t_{\text{CuO}x}\right)/j_{C}^{\text{CuO}x}\left(t_{\text{CuO}x}\right)$$ (3)

thereby defining a temperature-modulation efficiency associated with the orbital contribution.

Notably, $\eta^{\text{CuO}x}$ exhibits a peak at *t*_CuO*x*_ ∼ 6 to 7 nm (Fig. 3F), indicating an optimal thickness scale for the orbital-mediated response. If the signal originated from bulk orbital transport (e.g., the OHE), it would be expected to increase with thickness and eventually saturate over a scale determined by the propagation length (*17*, *42*). The pronounced peak instead suggests an interfacial origin of OAM generation, consistent with an OREE-driven SPE heat-modulation mechanism. Considering an estimated oxide-layer thickness of ∼2 to 3 nm (Fig. 3A), OAM generation is likely most effective when oxidized and metallic Cu regions coexist, a configuration reported to favor the OREE (*15*, *29*, *34*, *35*, *43*–*46*). First-principles calculations have shown that hybridization between O 2p and Cu 3d orbitals induces a pronounced orbital Rashba effect in surface-oxidized Cu (*35*). More recently, combined x-ray photoelectron spectroscopy (XPS), transmission electron microscopy (TEM), electron energy-loss spectroscopy (EELS), and first-principles analyses identified orbital hybridization at the Cu_4_O_3_/Cu interface as a key factor in enhancing orbital-current generation in naturally oxidized CuO*_x_* heterostructures (*46*). These findings support our interpretation that the coexistence of oxidized and metallic Cu regions promotes interfacial OAM generation.

At this optimal thickness, the peak value (177 × 10^−14^ K·m^2^/A) exceeds the conventional spin-driven SPE value measured in the same device (58 × 10^−14^ K·m^2^/A) and the representative value reported for YIG/Pt (47 × 10^−14^ K·m^2^/A) (*41*), highlighting efficient charge-to-heat conversion mediated by the OAM. This wedge-based approach minimizes uncertainties arising from batch-to-batch variations and interfacial differences, enabling direct evaluation of the intrinsic thickness dependence. At larger *t*_CuO*x*_, the signal monotonically decreases and becomes comparable to the experimental uncertainties. The orbital contribution persists into the thickness regime where a direct Pt/CuO*_x_* interface is effectively suppressed, further suggesting that the signal is associated with the Cu/CuO*_x_* interface. To further quantify this behavior, we fitted the decreasing data for *t*_CuO*x*_ ≥ 7 nm using an exponential function $\text{exp}\left(-\frac{t_{\text{CuO}x}}{\lambda_{\text{eff}}^{\text{CuO}x}}\right)$, where $\lambda_{\text{eff}}^{\text{CuO}x}$ represents an effective characteristic length. The decay is well described by the exponential function within the experimental uncertainty (*R*^2^ > 0.996), yielding $\lambda_{\text{eff}}^{\text{CuO}x}$ = 11.0 ± 0.5 nm. This exponential decay indicates a diffusion-like attenuation of the interfacial orbital contribution. The extracted length scale $\lambda_{\text{eff}}^{\text{CuO}x}$ = 11.0 ± 0.5 nm is comparable to the orbital transport length scale reported in a previous study on metallic Cu, where a characteristic length of ∼9 nm was obtained from time-resolved measurements (*47*). The observed behavior strongly supports OAM generation at the Cu/CuO*_x_* interface, whereas a direct Pt/CuO*_x_* contribution is unlikely, with the signal attenuating through the metallic Cu layer between Pt and CuO*_x_*. As an independent check, a finite $\eta^{\text{CuO}x}$ was also obtained for a fixed-thickness isolated-line device, indicating that the orbital contribution persists without the continuous wedge geometry (fig. S5C).

### Origin of the orbital-current-induced temperature modulation

To further investigate the origin of OAM generation, we fabricated additional Al-capped and Al-inserted samples using the same procedures (see Materials and Methods), as shown in Fig. 4 (A to C). The Al-capped sample [YIG/Pt(2.5)/Cu(*t*_CuO*x*_)/Al(2), where all thicknesses are in nanometers] was fabricated to suppress oxidation of Cu and thereby inhibit the formation of CuO*_x_*. The Al-inserted samples [YIG/Pt(2.5)/Al(1 or 2)/CuO*_x_*(*t*_CuO*x*_)] were fabricated to probe the origin of the signals by suppressing the Pt/CuO*_x_* interface while preserving the Cu/CuO*_x_* interface. Ultrathin Al layers (with a thickness of ∼0.8 nm) have been reported to provide appreciable interfacial orbital transparency (*48*). Therefore, if the OAM is generated at the Cu/CuO*_x_* interface via the OREE and propagates toward Pt as an orbital current through the metallic Cu layer, as suggested by the results in Fig. 3, this Al-inserted structure is expected to exhibit a finite orbital-driven temperature modulation. The patterned lines were oriented along the *x* direction, perpendicular to the *t*_CuO*x*_-gradient direction, enabling discrete evaluation at selected thicknesses. Four patterned lines, L1 to L4, were fabricated corresponding to *t*_CuO*x*_ = 0, 0.8, 2.9, and 5.6 nm, respectively. The variation in *t*_CuO*x*_ across the width of each patterned line was estimated to be ∼0.3 nm and is represented by the horizontal error bars in Fig. 4D. L1 was used as a reference, and L2 (*t*_CuO*x*_ = 0.8 nm) exhibited nearly identical signals. All LIT measurements were conducted sequentially by attaching electrodes to each patterned line. Using the LIT measurements and quantitative signal separation described above, we extracted the orbital contribution η^CuO*x*^ (Fig. 4D). The detailed LIT data are provided in fig. S6.

**Fig. 4.**
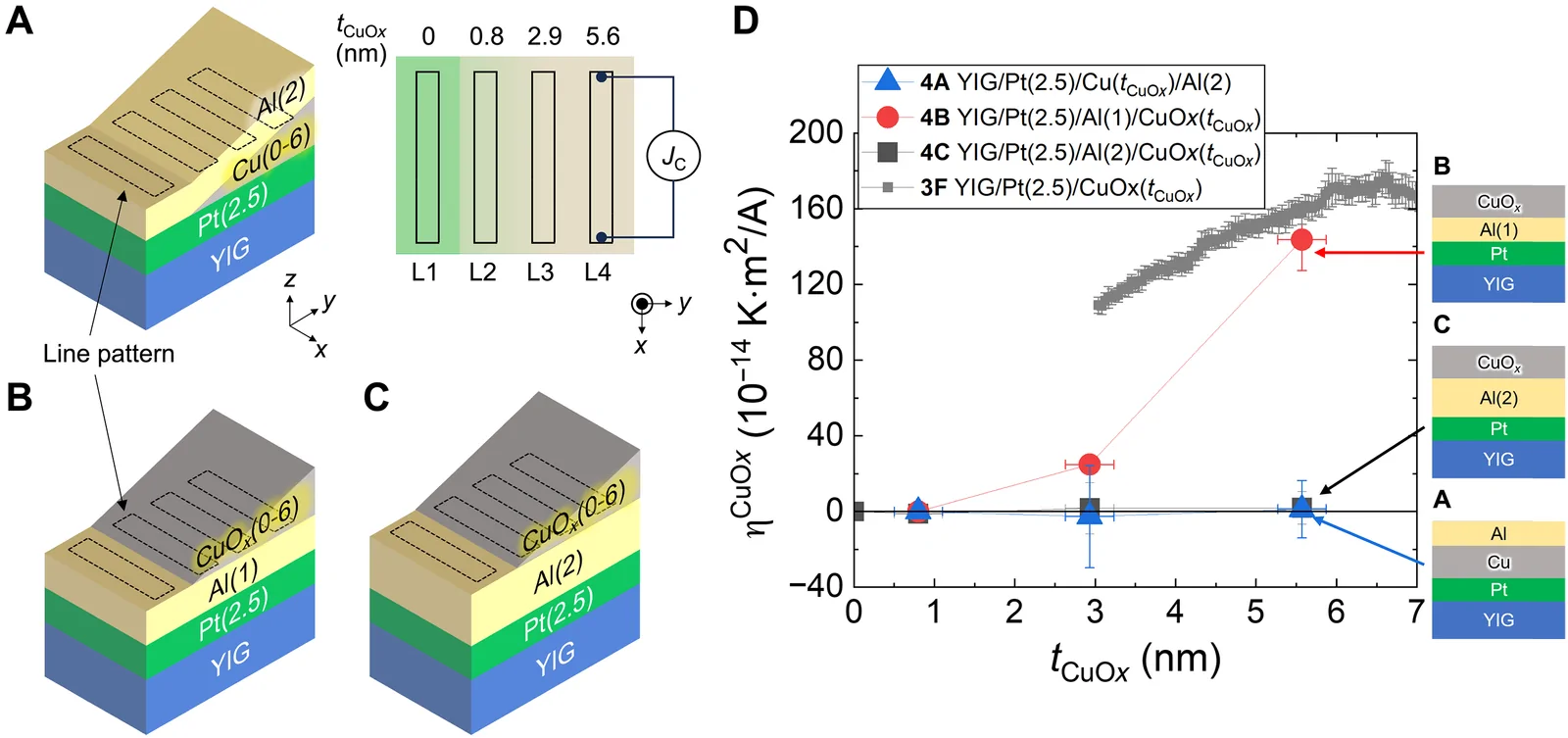
Separated orbital-angular-momentum-induced temperature modulation efficiency in samples patterned along the *x* direction, perpendicular to the CuO*_x_* thickness gradient. (**A** to **C**) Schematic illustrations of sample structures with (A) YIG/Pt(2.5 nm)/Cu(*t*_CuO*x*_) capped with a 2-nm-thick Al layer to suppress surface oxidation, (B) YIG/Pt(2.5 nm)/Al(1 nm)/CuO*_x_*(*t*_CuO*x*_), and (C) YIG/Pt(2.5 nm)/Al(2 nm)/CuO*_x_*(*t*_CuO*x*_), where a thin Al layer is inserted between the Pt and CuO*_x_* layers. (**D**) Orbital temperature modulation efficiency η^CuO*x*^ for the samples in (A) to (C) and the main data in Fig. 3F for reference. The corresponding LIT images and analysis regions are provided in fig. S6.

The Al-capped sample [YIG/Pt(2.5)/Cu(*t*_CuO*x*_)/Al(2); Fig. 4A] showed negligible orbital contribution (∼2 × 10^−14^ K·m^2^/A) for both L3 and L4, well below the experimental uncertainty (4 × 10^−14^ to 6 × 10^−14^ K·m^2^/A). In these samples, the observed signal is dominated by the conventional SHE in Pt. This result indicates that, in the YIG/Pt/CuO*_x_* samples used for the experiments in Figs. 2 and 3, CuO*_x_* plays a critical role in OAM generation through the OREE, consistent with previous theoretical and experimental studies (*15*, *29*, *34*, *35*, *43*–*45*). Although bulk orbital currents generated in Cu have been reported (*49*), the disappearance of the signal upon Al capping indicates that bulk contributions are not the dominant origin of the temperature modulation observed in our system. It is worth noting that both YIG/Cu(5)/Pt(5) and YIG/Al_2_O_3_(1)/Pt(5) produce negligibly small SPE signals (*41*), further indicating that pure Cu or oxidized Al layers alone do not produce a measurable SPE response in the present configuration.

We then investigated structures with an Al layer inserted between Pt and CuO*_x_* (Fig. 4, B and C), designed to disrupt the Pt/CuO*_x_* interface while preserving the Cu/CuO*_x_* side of the stack. With a 1-nm-thick Al layer, which is reported to provide appreciable interfacial orbital transparency (*48*), we observed a finite orbital contribution with a maximum η^CuO*x*^ of 144 × 10^−14^ K·m^2^/A at L4. However, no orbital-driven signal was observed when the Al thickness was 2 nm. The persistence of the signal with the 1-nm-thick Al insertion, despite disruption of the direct Pt/CuO*_x_* interface, supports OAM generation at the Cu/CuO*_x_* interface (*46*, *50*). Its disappearance with the 2-nm-thick Al insertion is consistent with reduced orbital-current transmission through the thicker Al layer. Together with the exponential decay trend observed in Fig. 3, these results strongly support the conclusion that OAM generation predominantly occurs at the Cu/CuO*_x_* interface, whereas contributions from a direct Pt/CuO*_x_* interface are unlikely in the present configuration.

## DISCUSSION

We demonstrate that orbital currents can contribute to temperature modulation in magnetic heterostructures via the SPE. Using YIG/Pt/CuO*_x_* trilayers with continuously tuned CuO*_x_* thickness, we identify an orbital-current-mediated contribution that becomes pronounced under optimized conditions. The thickness-dependent behavior, characterized by a distinct maximum followed by exponential decay, together with control experiments using Al-capped and Al-inserted structures, indicates that the observed temperature modulation is primarily associated with interfacial OAM generation at the Cu/CuO*_x_* interface. The suppression of the orbital contribution in the Al-capped structure and the persistence of a finite signal with a 1-nm-thick Al insertion highlight the essential role of interfacial coupling, ruling out bulk orbital transport as the dominant mechanism. These results establish a direct link between charge-current-driven OAM transport and SPE-induced temperature modulation, extending the scope of spin caloritronics beyond spin-dominated frameworks. In addition, the interplay between orbital and spin currents suggests a potential driving principle for transverse thermoelectric conversion, where angular-momentum transport can be used for heat-to-charge energy conversion. In this context, orbital contributions may provide an additional degree of freedom to complement established spin-based mechanisms (*51*, *52*). Future observation of the orbital Peltier effect, the Onsager reciprocal counterpart of the orbital Seebeck effect, would further broaden the framework of orbital-mediated heat transport. The ability to engineer interfacial OAM generation and transport provides a design principle for heat control in solids, thereby opening a pathway toward spin-orbit caloritronics and highlighting the potential for further performance improvement in solid-state thermal management and energy conversion technologies.

## MATERIALS AND METHODS

### Sample preparation

The heterostructure samples were fabricated by depositing thin metallic layers using a magnetron sputtering system (Comet Inc., CMS-A6250X2) on (111)-oriented single-crystalline YIG grown on (111)-oriented GGG substrates by liquid-phase epitaxy. The GGG/YIG substrates were mirror-polished before deposition using a slurry containing 50-nm-diameter alumina particles (Baikowski International Corporation) and cleaned by sonication in acetone and deionized water to remove residues. dc power sources were used for the Pt, Cu, and Al targets with a power of 30 W, and the deposition rates were 0.0349, 0.0339, and 0.0123 nm/s, respectively. The deposition was conducted at room temperature under vacuum conditions with a base pressure below 6.0 × 10^−6^ Pa and at an Ar process gas pressure of 0.5 Pa. Uniform films of Pt and Al were deposited while rotating the substrates, whereas the wedged Cu layer was fabricated using a linearly moving shutter over a length of ∼6 mm at a fixed substrate angle. The layers were deposited without breaking vacuum. All samples were exposed to ambient air for 2 days before further processing. In samples without a capping layer, this exposure resulted in the formation of naturally oxidized CuO*_x_* layers from the deposited Cu films, whereas the Al-capped sample was protected from oxidation. For the LIT samples, electrical contacts were formed at the edges of the patterned lines using silver epoxy, which was cured at 80°C for 30 min. The samples used for electrical resistivity measurements were subjected to the same thermal treatment before measurement.

### LIT measurement and field antisymmetrization

The spatial distribution of temperature modulation associated with the SPE was measured using a LIT system (DCG Systems Inc., ELITE). Before the LIT measurements, all sample surfaces were uniformly coated with black ink having an emissivity greater than 0.94. For the continuous wedge sample shown in Fig. 2, a 1× infrared lens with an *f*-number of 1.5 and a working distance of 40 mm was used, corresponding to a pixel size of 15 × 15 μm^2^. The total image dimensions were 329 × 486 pixels, and the quantitative analysis was performed over a region of 17 × 486 pixels. For the measurements corresponding to Fig. 4 and shown in fig. S6, a 5× infrared lens with an *f*-number of 4.0 and a working distance of 14 mm was used, corresponding to a pixel size of 3 × 3 μm^2^. The total image dimensions were 446 × 304 pixels, and the quantitative analysis was performed over a region of 91 × 304 pixels.

To isolate the magnetization-dependent SPE component, the signals were antisymmetrized with respect to the magnetic field (±*H*). The complex LIT signal measured under each magnetic-field direction was defined as

$$Z_{\pm H}=A_{\pm H}\text{exp}\left(-i\phi_{\pm H}\right)$$ (4)

where $A_{\pm H}$ and $\phi_{\pm H}$ denote the lock-in amplitude and phase measured under positive or negative *H*, respectively. The field-odd complex LIT signal was obtained by complex antisymmetrization as follows (*11*, *39*, *40*)

$$Z_{\text{odd}}=\frac{Z_{+H}-Z_{-H}}{2}$$ (5A)

The corresponding field-odd amplitude and phase, *A*_odd_ and ϕ_odd_, were determined from

$$A_{\text{odd}}=|Z_{\text{odd}}|=\left|\frac{A_{+H}\text{exp}\left(-i\phi_{+H}\right)-A_{-H}\text{exp}\left(-i\phi_{-H}\right)}{2}\right|$$ (5B)

$$\begin{array}{c} \phi_{\text{odd}}=-\text{arg}\left(Z_{\text{odd}}\right)\\ =-\text{arg}\left[\frac{A_{+H}\text{exp}\left(-i\phi_{+H}\right)-A_{-H}\text{exp}\left(-i\phi_{-H}\right)}{2}\right] \end{array}$$ (5C)

Similarly, the field-even complex LIT signal was obtained by complex symmetrization

$$Z_{\text{even}}=\frac{Z_{+H}+Z_{-H}}{2}$$ (6A)

The corresponding field-even amplitude and phase, *A*_even_ and ϕ_even_, were determined from

$$A_{\text{even}}=|Z_{\text{even}}|=\left|\frac{A_{+H}\text{exp}\left(-i\phi_{+H}\right)+A_{-H}\text{exp}\left(-i\phi_{-H}\right)}{2}\right|$$ (6B)

$$\begin{array}{c} \phi_{\text{even}}=-\text{arg}\left(Z_{\text{even}}\right)\\ =-\text{arg}\left[\frac{A_{+H}\text{exp}\left(-i\phi_{+H}\right)+A_{-H}\text{exp}\left(-i\phi_{-H}\right)}{2}\right] \end{array}$$ (6C)

Because the conventional Peltier effect is independent of *H* and the magneto-Peltier effect is symmetric with respect to *H*, their contributions do not appear in the field-odd amplitude and phase, *A*_odd_ and $\phi_{\text{odd}}$.

### Electrical transport measurements

The resistivity was calculated from the measured resistance using the geometrical dimensions of the channel at room temperature. The distance between the voltage electrodes was 4.4 mm, and the channel width was 0.3 mm. The samples for electrical resistivity estimation were fabricated using the same procedure as the main samples (described above). A standard four-probe method was used with a digital multimeter (Keithley, DMM6500) and a probe system equipped with four needle probes. The measured voltage fluctuation was less than 0.1% for all samples.

For the time-dependent electrical characterization, the resistance of a YIG/Pt(2.5 nm)/Cu(6 nm) reference sample was measured in a four-probe configuration using the same digital multimeter. The measurement was initiated within 20 min after the sample was removed from the sputtering chamber and was performed at 10-min intervals. The sample was maintained under ambient conditions for 48 hours and subsequently heated on a hot plate at 80°C for 30 min to reproduce the silver-epoxy curing treatment used for the LIT samples. After the sample cooled to room temperature, resistance monitoring was resumed for a further 24 hours.
