# Supplementary material for: Observation of orbital-angular-momentum-driven temperature modulation via the spin Peltier effect
Source: Sci Adv. 2026 Oct 2;12(40):eaej1838. doi: 10.1126/sciadv.aej1838 (PMC13632755; doi:10.1126/sciadv.aej1838)
Supplement: Supplementary file 1 [file sciadv.aej1838_sm.pdf]

Science Advances
AAAS

Supplementary Materials for

Observation of orbital-angular-momentum-driven temperature modulation via the spin Peltier effect

Sang J. Park *et al.*

Corresponding author: Sang J. Park, sangjunpark94@gmail.com; Ken-ichi Uchida, UCHIDA.Kenichi@nims.go.jp

Sci. Adv. **12**, eaej1838 (2026)
DOI: 10.1126/sciadv.aej1838

This PDF file includes:

Figs. S1 to S6

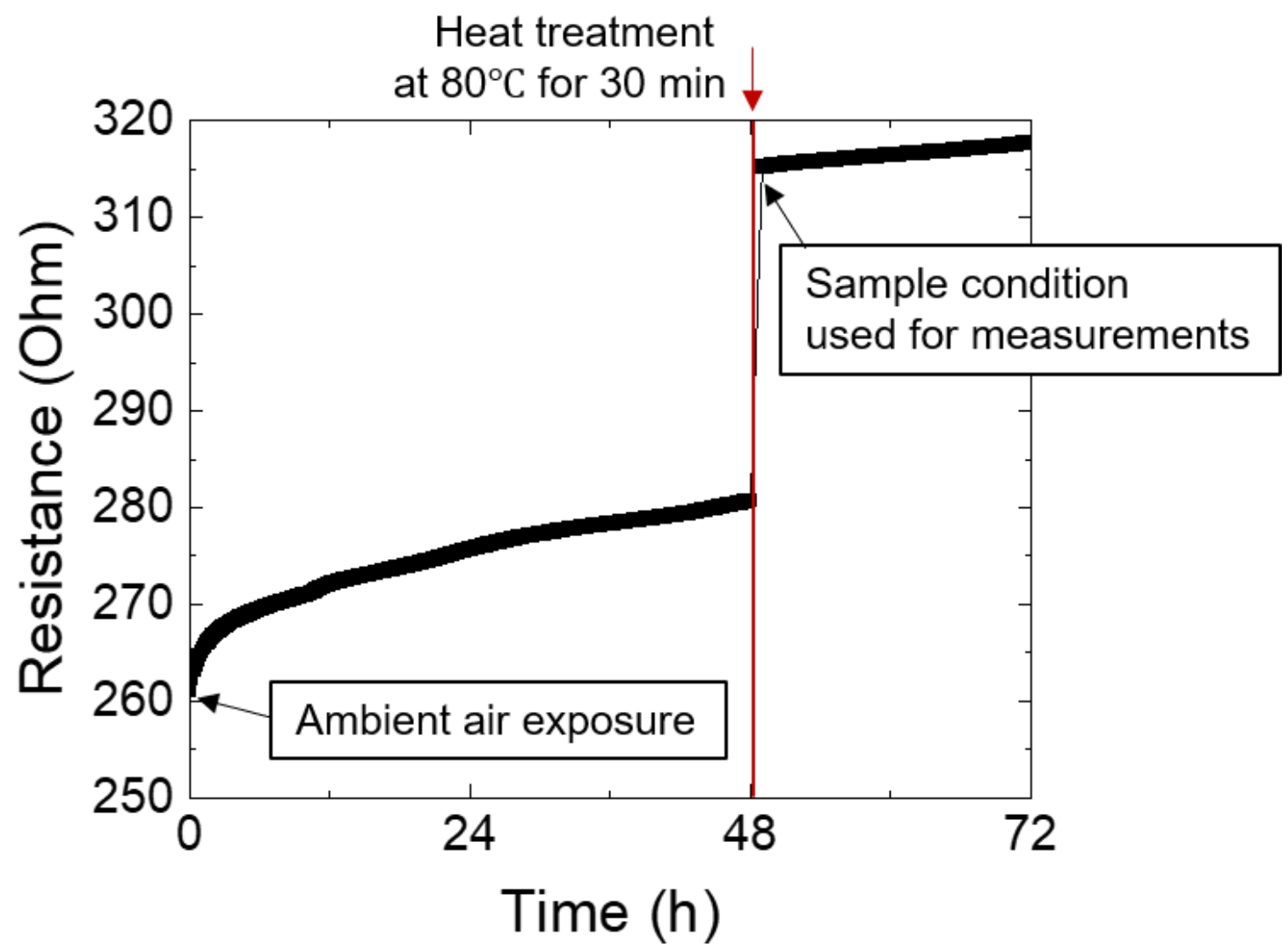

Fig. S1. Time-dependent resistance of a YIG/Pt(2.5 nm)/Cu(6 nm) reference sample. The resistance measurement was initiated within 20 min after the sample was removed from the sputtering chamber and was performed at 10-min intervals. The sample was maintained under ambient conditions for 48 h and subsequently heated at 80℃ for 30 min to reproduce the silver-epoxy curing treatment. After the sample cooled to room temperature, resistance monitoring was resumed for a further 24 h. The resistance increased rapidly during the initial ambient exposure, particularly during the first 5 h, and subsequently increased at a reduced rate. Following the 80°C treatment, a further resistance change occurred, and the resistance continued to increase slightly during the subsequent 24 h at room temperature.

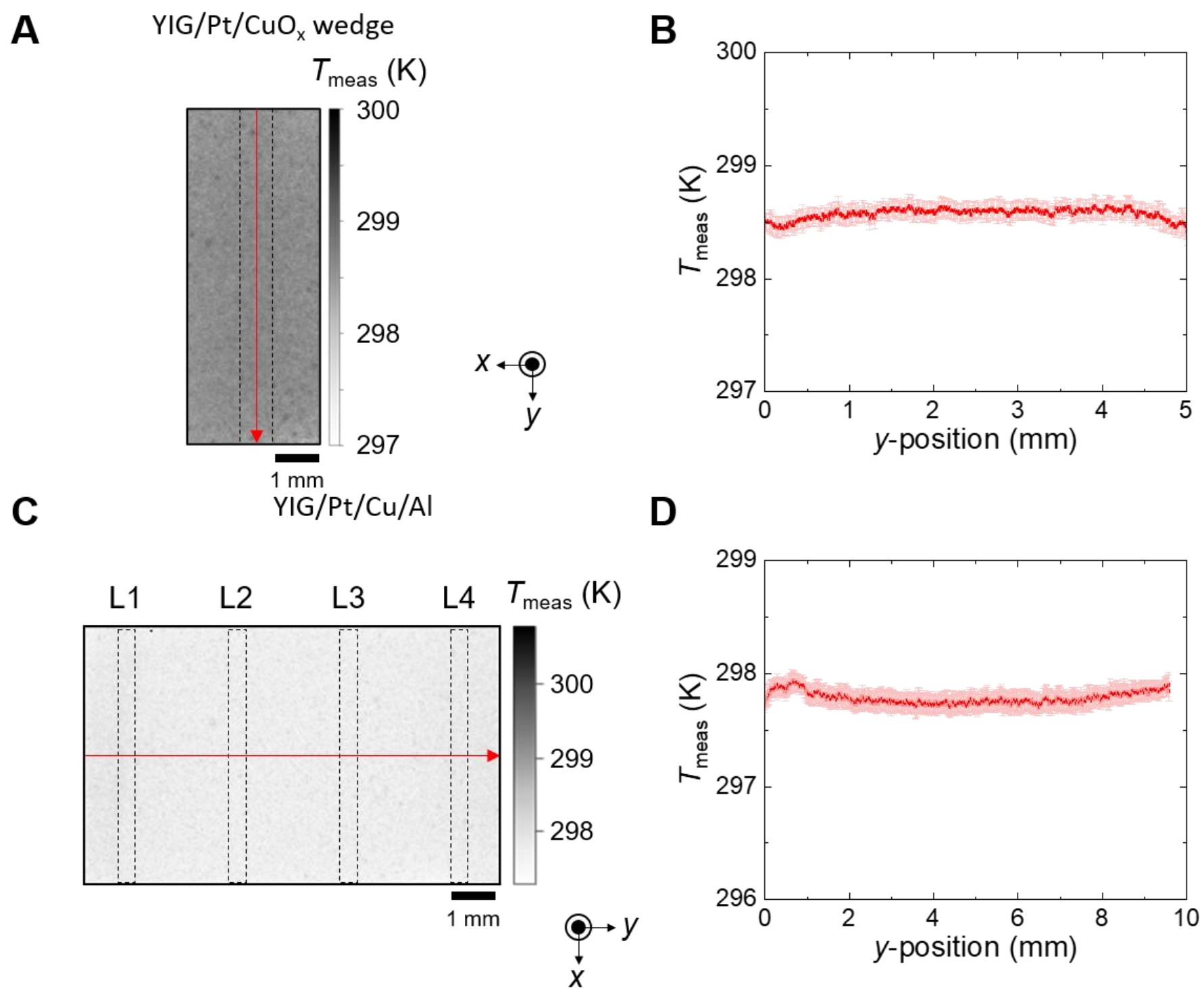

Fig. S2. Steady-state surface-temperature distributions measured without an applied charge current. (A) Surface-temperature map of the YIG/Pt/CuO_x wedge sample, corresponding to Fig. 2 in the main text. (B) Corresponding line profile along the *y* direction indicated by the red arrow in (A). (C) Surface-temperature map of the YIG/Pt/Cu/Al sample, corresponding to Fig. 4 in the main text. (D) Corresponding line profile along the *y* direction indicated by the red arrow in (C). The white dashed boxes indicate the sample regions. No apparent temperature contrast was observed between the sample regions and the surrounding areas, supporting sufficiently uniform infrared emissivity across the sample surfaces under the present measurement conditions. The images were acquired using a 1× infrared lens.

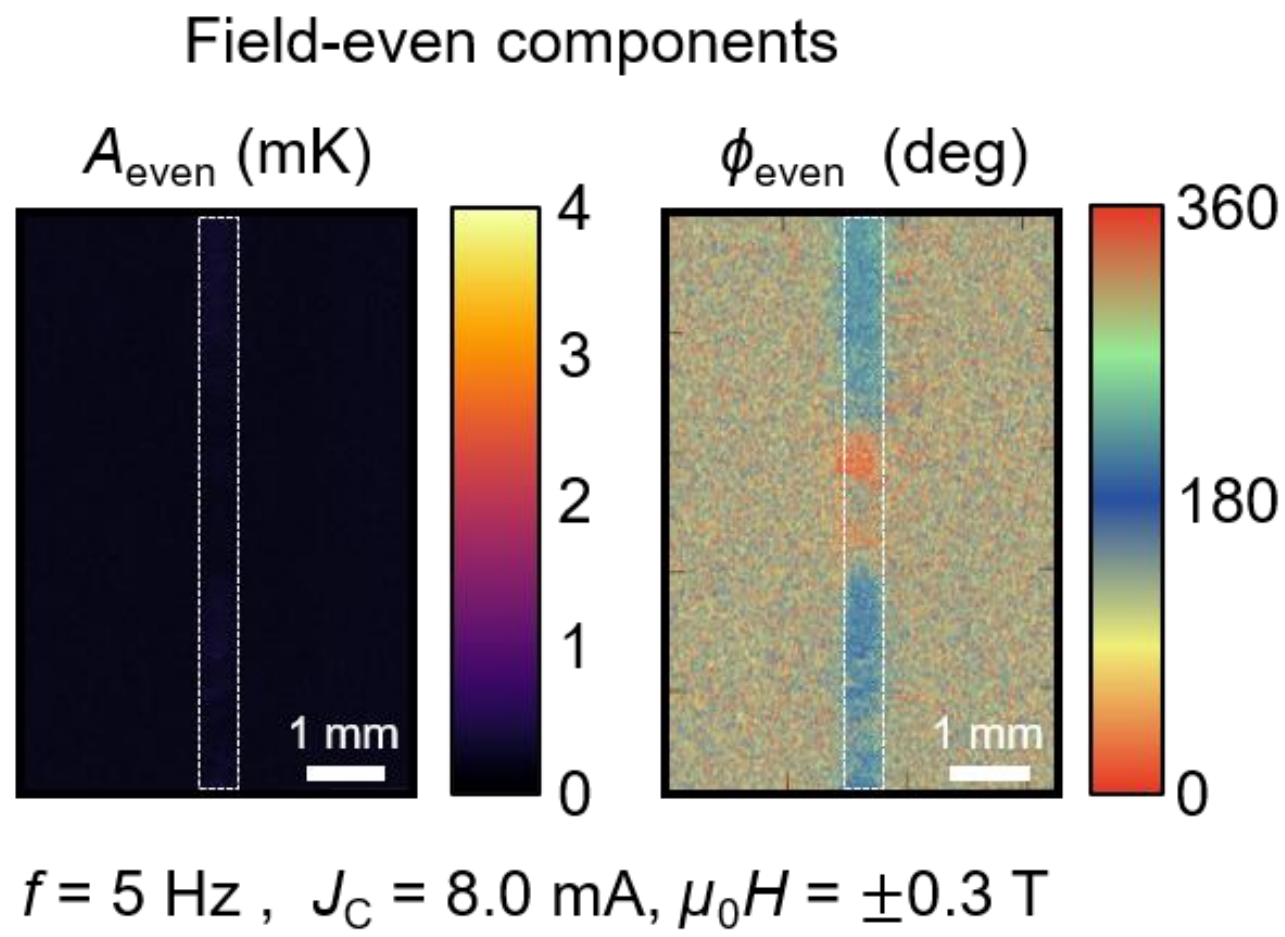

Fig. S3. Field-even lock-in amplitude (A_{even}) and phase (ϕ_{even}) images of the YIG/Pt (2.5 nm)/CuO_x wedge (0–16 nm) sample. Definitions are given in the MATERIALS AND METHODS. The same color scales as those in Fig. 2 of the main text were used. The white dashed boxes indicate the sample outline and are provided as guides to the eye. The A_{even} values are comparable to the background fluctuation level and are typically less than 0.2 mK.

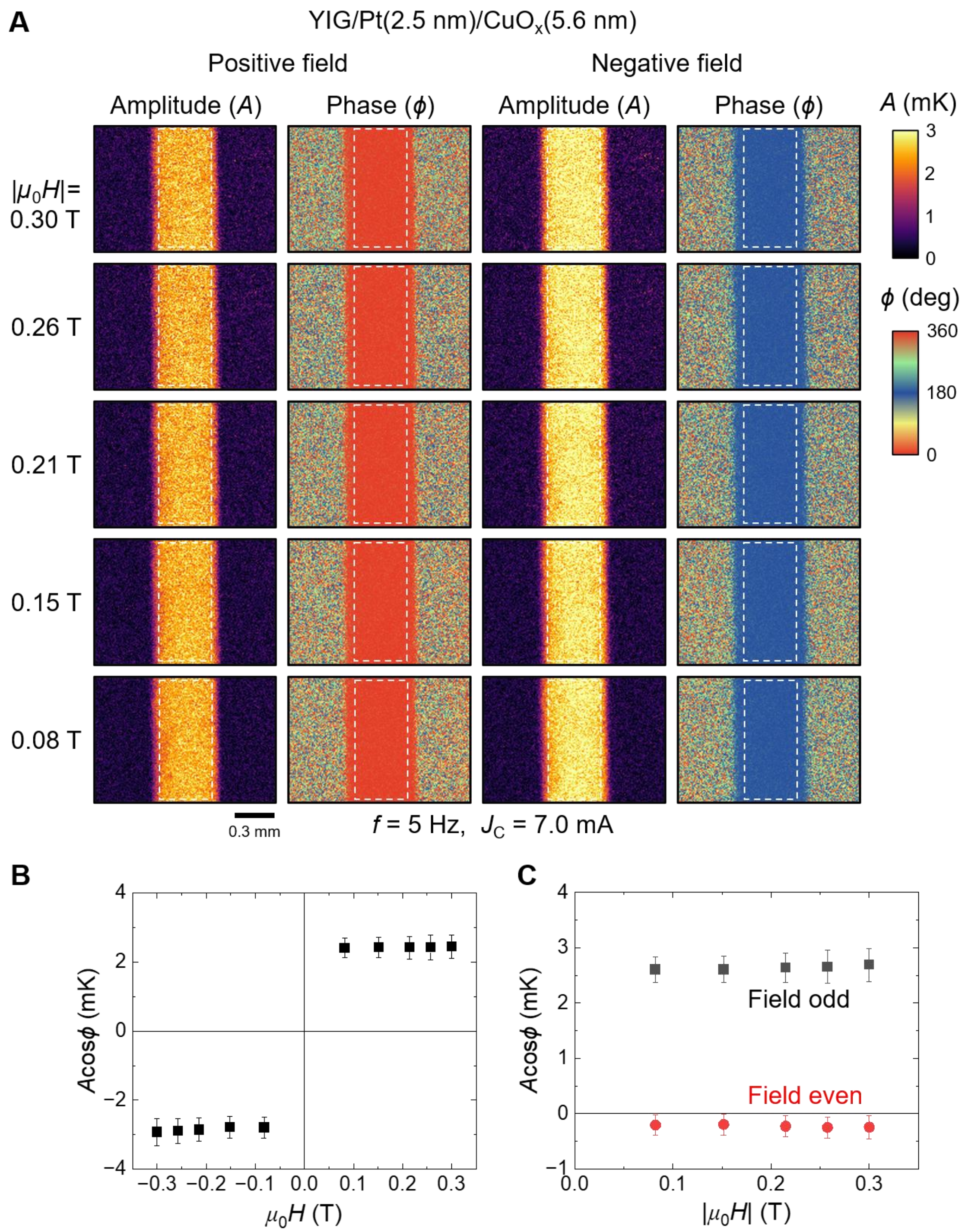

Fig. S4. Magnetic-field dependence of the LIT signal in a YIG/Pt(2.5 nm)/CuO_x(5.6 nm) device. (A) Raw lock-in amplitude (A) and phase (ϕ) measured at each magnetic field (μ_0H). The measurements were performed at a fixed frequency of f = 5 Hz and an applied charge current of J_C = 7.0 mA. (B) Magnetic-field dependence of the in-phase temperature-modulation component, $A\cos\phi$. (C) Field-odd and field-even components obtained by antisymmetrization and symmetrization of the complex LIT signal. The LIT images were acquired using a 5× infrared lens.

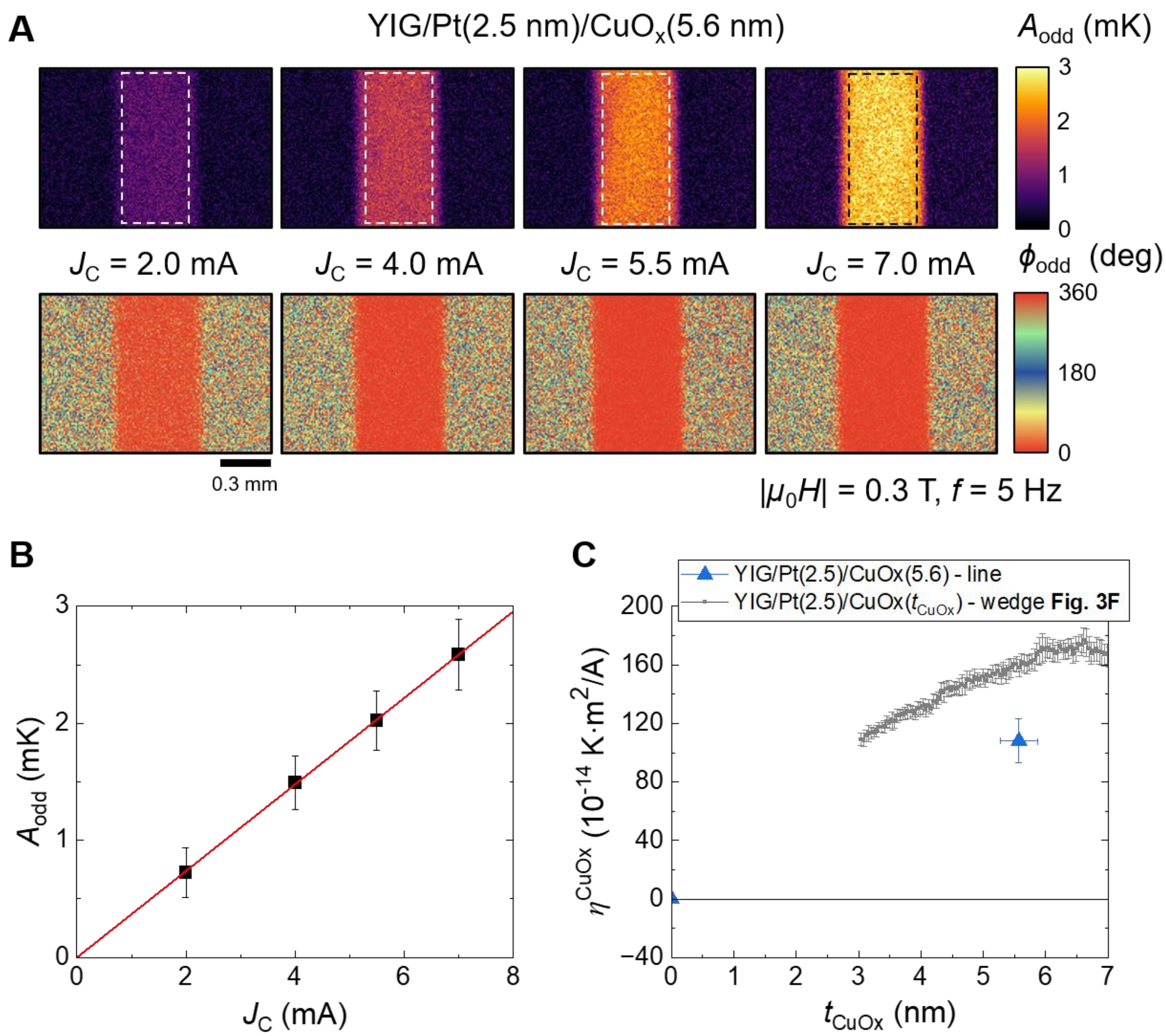

Fig. S5. Applied-current dependence of the LIT signal in a YIG/Pt(2.5 nm)/CuO_x(5.6 nm) device. (A) Field-odd lock-in amplitude (A_{odd}) and phase (ϕ_{odd}) measured as a function of the applied charge current (J_C). The measurements were performed at a fixed frequency of $f = 5$ Hz and opposite magnetic fields $\mu_0 H = \pm 0.3$ T. (B) Applied-current dependence of A_{odd}. (C) Charge-to-heat conversion efficiency η^{CuOx} associated with the CuO_x orbital contribution, defined as the SPE-induced temperature modulation normalized by the current density flowing through the CuO_x layer (see Equation 3 in the main text). The values for the wedge sample are replotted from Fig. 3F for comparison. The LIT images were acquired using a 5× infrared lens.

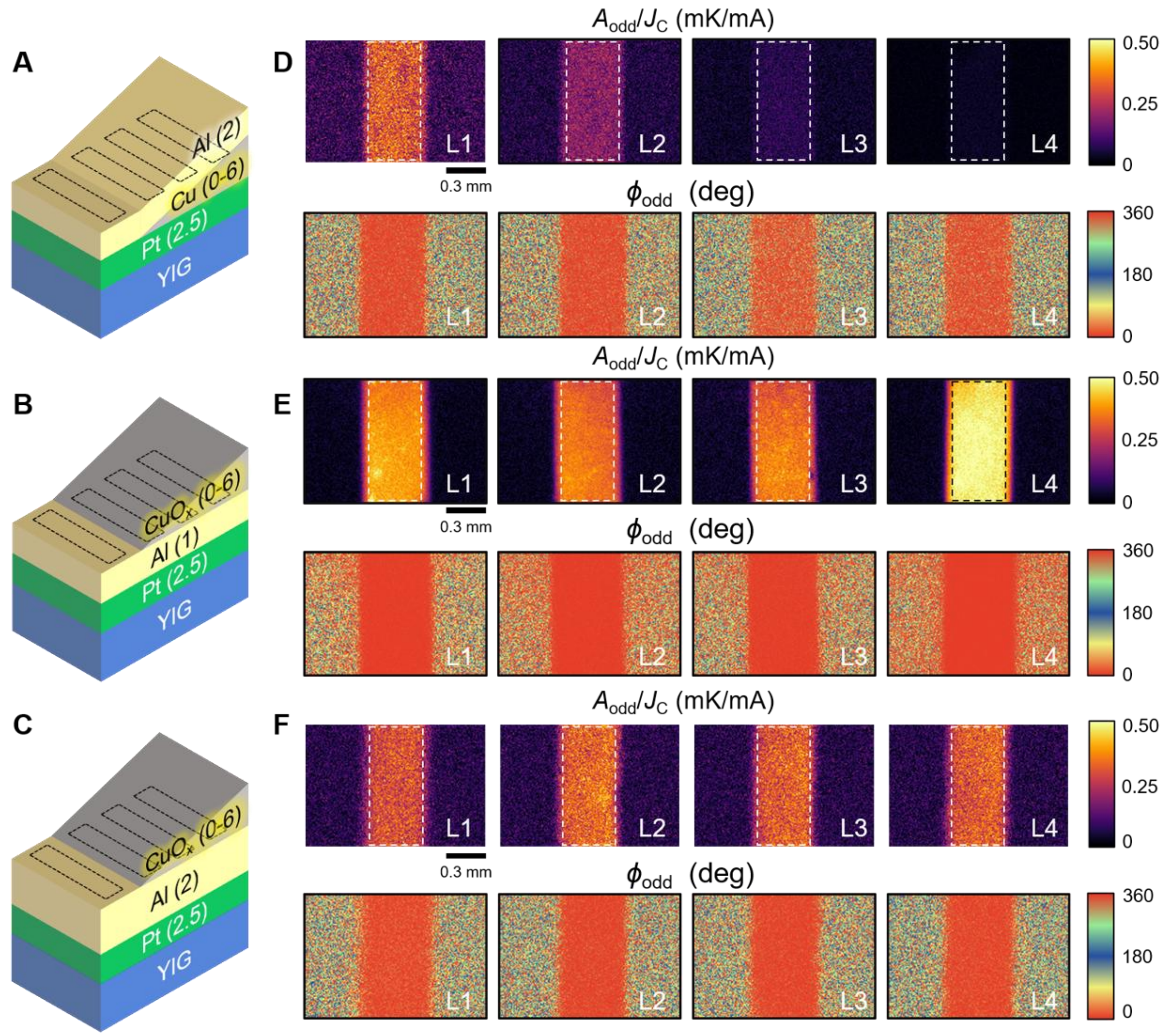

Fig. S6. Schematic illustrations of the samples and measured lock-in thermography data. (A)–(C) Schematic illustrations of the samples with (A) YIG/Pt(2.5 nm)/Cu(0–6 nm, wedge)/Al(2 nm), (B) YIG/ Pt(2.5 nm)/Al(1 nm)/CuO_x(0–6 nm, wedge), and (C) YIG/Pt(2.5 nm)/Al(2 nm)/CuO_x(0–6 nm, wedge), corresponding to Fig. 4A–C of the main text, respectively. (D)–(F) Field-odd lock-in amplitude (A_{odd}) per applied current J_C and phase (ϕ_{odd}) for the samples. The values were obtained by averaging over the boxed regions. The LIT images were acquired using a 5× infrared lens, corresponding to a pixel size of 3 × 3 μm^2. Additional imaging and analysis details are provided in the MATERIALS AND METHODS.
